# Development of an Illumina-based ChIP-exonuclease method provides insight into FoxA1-DNA binding properties

**DOI:** 10.1186/gb-2013-14-12-r147

**Published:** 2013-12-27

**Authors:** Aurelien A Serandour, Gordon D Brown, Joshua D Cohen, Jason S Carroll

**Affiliations:** 1 Cancer Research UK Cambridge Institute, University of Cambridge, Robinson Way, Cambridge CB2 0RE, UK

## Abstract

ChIP-exonuclease (ChIP-exo) is a modified ChIP-seq approach for high resolution mapping of transcription factor DNA sites. We describe an Illumina-based ChIP-exo method which provides a global improvement of the data quality of estrogen receptor (ER) ChIP and insights into the motif structure for key ER-associated factors. ChIP-exo of the ER pioneer factor FoxA1 identifies protected DNA with a predictable 8 bp overhang from the Forkhead motif, which we term mesas. We show that mesas occur in multiple cellular contexts and exist as single or overlapping motifs. Our Illumina-based ChIP-exo provides high resolution mapping of transcription factor binding sites.

## Background

Since the last decade, ChIP-on-chip and ChIP-seq technologies have considerably increased our understanding of the functional organization of the genome [1]. These technologies allow the genome-wide mapping of chromatin-associated proteins and histone marks. ChIP-seq is now commonly used to study a wide range of biological processes including transcription, replication, DNA repair and evolution of the genome [2,3]. ChIP-seq of transcription factors is particularly useful to determine their bound DNA motifs and target genes. Nevertheless the resolution of ChIP-seq is inadequate to resolve positional information between different motifs within binding sites; additionally, overlaps between different ChIP-seq datasets can be exaggerated due to the width of peaks. The precise determination of the bound DNA motifs and their positions relative to other motifs is of importance for understanding the features involved in transcription factor-DNA interactions, an important level of information when considering, for example, GWAS-identified single nucleotide polymorphisms (SNPs) within a transcription factor binding site [4].

Recent studies from Pugh and colleagues report a SOLiD platform-based method called ChIP-exonuclease (ChIP-exo), which greatly increases the resolution of ChIP peaks [5,6]. To date, these experiments have been mostly limited to yeast models. Due to the fact that the Illumina sequencing platform is currently the most common sequencing technology, we sought to adapt ChIP-exo from the SOLiD to the Illumina platform. We apply the Illumina-based ChIP-exo to human cancer cell line experiments and directly compare the resolution of the peaks to ChIP-seq. We find ChIP-exo to outperform ChIP-seq by all parameters, revealing unexpected insight into the FoxA1-DNA interface in breast cancer cells and in mouse liver.

## Results and discussion

### An Illumina-based ChIP-exo method

**Figure 1:**
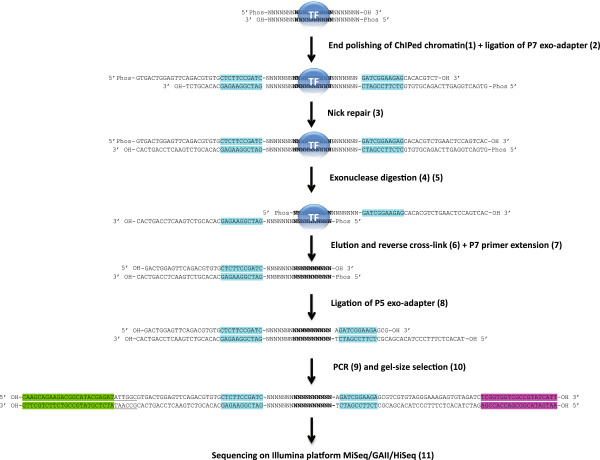
**Illustration of the Illumina-adapted ChIP-exo strategy.** To carry out ChIP-exo, the P5 adapter is ligated upstream and downstream of the exonuclease digestion-protected region. The ChIP-exo library is sequenced with single-end reads from the P5 adapter. The reads are mapped on the reference genome. The overlap between the reads mapped on the top and the bottom strands is considered as the exonuclease digestion-protected region. The index sequence is underlined. The P7 flow cell capture sequence is in green. The P5 flow cell capture sequence is in purple. The P5/P7 complementary sequence is in blue.

Our ChIP-exo method is derived from Pugh’s method [5]. This genome-wide mapping method is believed to increase the ChIP resolution by allowing the *lambda* exonuclease to digest the ChIPed DNA until the first point of cross-linking between the DNA and the ChIPed protein. In designing 18 different oligonucleotides (Figure 1 and Additional file 1: Table S1), we have been able to successfully adapt this method from the SOLiD to the Illumina sequencing platform including the MiSeq, GAIIx and Hiseq 2000/2500 sequencers. We have also been able to sequence and demultiplex successfully a pool of 12 ChIP-exo libraries, each of them having a different index sequence (Additional file 2: Figure S1 and Additional file 1: Table S2).

After an Illumina ChIP-seq library preparation, each ChIPed DNA fragment is ligated to the P7 and P5 adapters on both sides. The single-end sequencing of the ChIP-seq library results in two shifted populations of reads, one mapped on the top strand and the other mapped on the bottom strand (Additional file 3: Figure S2). These two shifted populations of reads are taken into consideration to estimate the centre of the peak using the peak caller MACS [7]. After our Illumina ChIP-exo library preparation, each ChIPed DNA fragment results in two library fragments: one with the P5 adapter ligated downstream of the exonuclease digestion-protected DNA and the other with the P5 adapter ligated upstream of it. In each case, the P7 adapter is ligated to the other extremity. The single-end sequencing of the ChIP-exo library results in two overlapping populations of reads, one mapped on the top strand and the other mapped on the bottom strand.

### Comparative analysis of ChIP-seq and ChIP-exo

**Figure 2:**
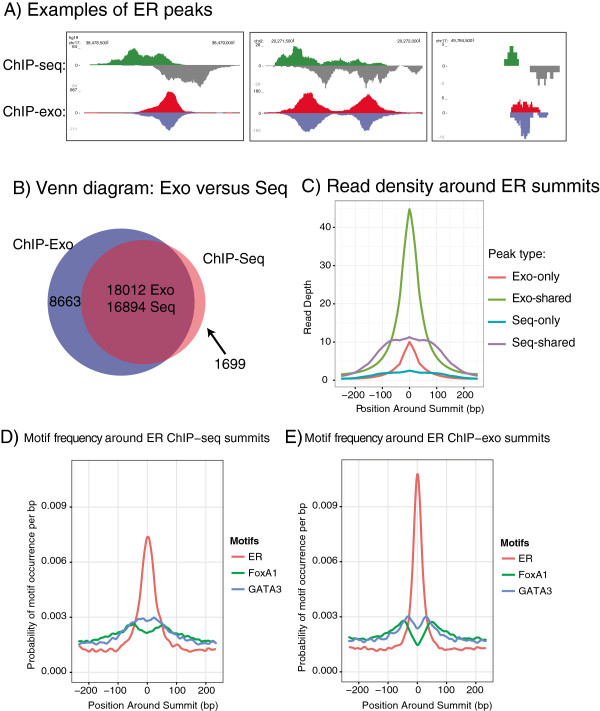
**Comparison of ChIP-exo with ChIP-Seq. (A)** Examples of ChIP-exo peaks and corresponding ChIP-seq peaks. **(B)** Venn diagram showing the overlap between consensus ChIP-exo peaks and consensus ChIP-seq peaks. The overlap region has separate numbers for -exo and -seq because some single peaks in -seq overlap two or more peaks in -exo. **(C)** Density plot of ER enrichment around summits, for exo-only, shared, and seq-only peaks. Shared peaks are shown separately for -exo and -seq peaks to show the difference in read depth and peak width. **(D)** Density plot of ER, FoxA1 and GATA3 motifs around ER summits found via ChIP-Seq. **(E)** Density plot of ER, FoxA1 and GATA3 motifs around ER summits found via ChIP-exo.

To directly compare ChIP-seq and our adapted ChIP-exo method for the Illumina sequencing platform, we mapped estrogen receptor α (ER) in human MCF-7 breast cancer cells by both methods. Three replicates each of ChIP-exo and ChIP-seq on ER were constructed from matched material. Each library was sequenced to a depth of approximately 10 million reads (Additional file 1: Table S3). Figures 2A and Additional file 4: Figure S3 show a comparison of example ER binding peaks. Note that the characteristic offset of top- and bottom-strand reads seen in ChIP-seq is not present in ChIP-exo, making analysis simpler, because there is no longer a requirement to estimate insert size and adjust the positive and negative strand reads accordingly. This can allow smaller peaks to be detected more reliably. Examples in Additional file 5: Figure S4 show that it is possible to discriminate between adjacent yet distinct binding events with ChIP-exo, which is generally not possible with ChIP-seq. Figure 2B shows the overlap between consensus ChIP-exo peaks and consensus ChIP-seq peaks. These are peaks that were found in all three replicates of ChIP-exo, or all three replicates of ChIP-seq. Most peaks that are only in the -exo libraries or only in the -seq libraries are weaker peaks; there is no evidence of systematic bias in peak detection. Figure 2C shows a density plot of mean enrichment around the peak summits: enrichment of -exo peaks is clearly stronger, even after normalizing for the number of reads. For peaks identified using ChIP-exo, reads cluster closer to the summit, making peak calling more reliable. Additional file 6: Figure S5A shows motif enrichment in each of the libraries; the rate of motif occurrence is higher in exo, even in the exo-only peaks, showing that these are likely real binding loci. Additional file 6: Figure S5B shows that motifs in the exo-only peaks have *p*-values broadly similar to the shared peaks, indicating that the exo-only peaks are less likely to be false positives than the seq-only peaks. In summary we find that ChIP-exo produces more reliable and robust results than ChIP-seq, with higher binding resolution and the discovery of peaks, missed by ChIP-seq, that have the hallmarks of bona fide transcription factor binding sites. ChIP efficiency, measured by the ratio of reads in peaks to total read count, is higher for ChIP-exo than ChIP-seq (Additional file 1: Table S4). Variability among replicates is roughly similar between ChIP-exo and ChIP-seq (Additional file 7: Figure S6), possibly indicating that the variations are normal biological variability rather than technical differences between the methods.

Using ChIP-seq, it has been challenging to resolve structure between functionally related transcription factors that bind to adjacent sequences and operate as a complex. For example, three key transcription factors involved in ER-DNA interactions are ER, FoxA1 and GATA3 [8,9]. Using the higher resolution of ChIP-exo, we measured the density of ER, FoxA1 and GATA3 motifs around ER summits. Figure 2D and 2E show the density of ER/FoxA1/GATA3 motifs around the ER seq-summits and ER exo-summits. The ChIP-exo ER motif density distribution is narrower than that of ChIP-seq with characteristic widths of 88 bp and 114 bp, respectively (see Methods), indicating ChIP-exo peak summits are called more consistently near the locations of transcription factor binding sites. Additionally, in both ChIP-seq and ChIP-exo, the ER, FoxA1 and GATA3 motifs are enriched near the ER summits, but the increased resolution of ChIP-exo peak summits affords clearer appreciation of how the transcription factors associate: namely GATA3 motifs are adjacent to the central ERE and further away from the GATA motifs are the Forkhead motifs (representing FoxA1 binding domains). This pattern appears to show a predictable structure that these three key breast cancer factors form in defining a transcriptionally active *cis*-regulatory element, a finding revealed by the increased resolution derived from ChIP-exo.

### Insights into transcription factor binding

**Figure 3:**
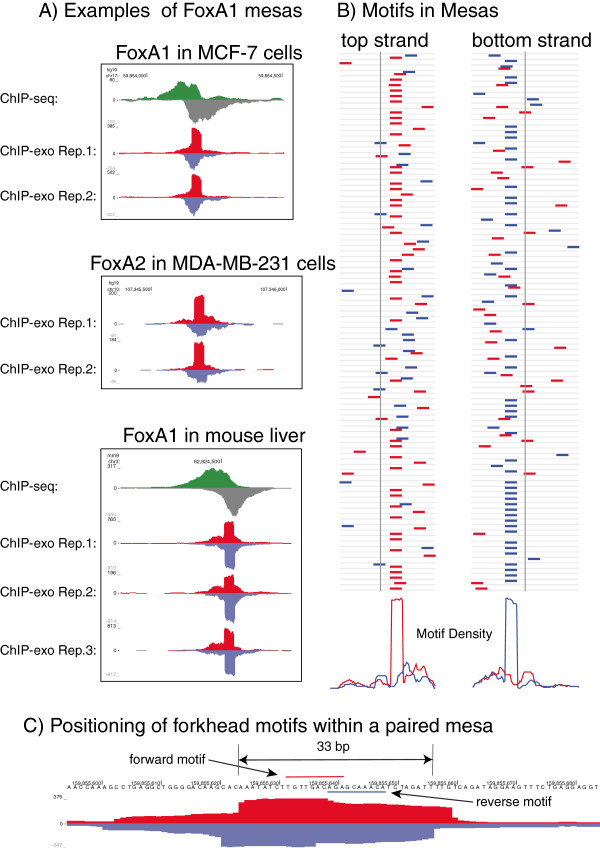
**Insights into transcription factor binding. (A)** Examples of a consistent increase in read depth at a single position, indicating that exonuclease digestion is blocked at this position. (The corresponding drop in read depth is simply a reflection of the 36-nucleotide reads from the sequencer.) The corresponding peaks from ChIP-seq are also shown for the FoxA1 examples. **(B)** A random sample of positions in the genome, 100 on the top strand and 100 on the bottom strand, at which the read depth increases by at least 100 reads on the top and bottom strands, respectively. The vertical line indicates the position of the increase in read depth. The red and blue lines indicate the position of top- and bottom-strand forkhead motifs, with a strong pattern of motifs exactly 9 bp downstream from the increase in read depth. The plot shows a window of 100 bp centred on the increase. The density plots below show the density of top (red) and bottom (blue) strand motifs across the window. **(C)** An example of paired mesas on the top and bottom strand, with overlapping motifs in a palindromic orientation. The 33 bp distance between the top- and bottom-strand mesa edges is the most common distance.

We utilised ChIP-exo to explore binding of other transcription factors, focusing on the ER associated pioneer factor FoxA1 [8,10-12]. When FoxA1 ChIP-exo was conducted we identified numerous peaks that showed a sharp accumulation of reads that occurred at precisely the same genomic location. This implies a stable FoxA1-DNA interface with predictable protection from enzymatic digestion from the exonuclease. Figure 3A shows an example of a sudden increase in read depth at a particular position; this pattern occurs in several thousand positions across the genome. We describe these regions as ‘mesas’ due to their resemblance to the geological features. We detect mesas in FoxA2 ChIP-exo conducted in ER negative/FoxA1 negative MDA-MB-231 cells and in FoxA1 ChIP-exo conducted in primary mouse liver. This suggests that the mesa digestion profile is conserved between FoxA1 and FoxA2, and between mammals. Analysis of the position of the Forkhead motif sequencing within the mesas revealed unexpected predictability in the relative location and direction of the motif, based on the edge of the protected regions of DNA. Figure 3B shows, for 100 randomly chosen top-strand and bottom-strand mesa leading edges, the position of top-strand (red) and bottom-strand (blue) forkhead motifs. The high frequency of motifs exactly 9 bp downstream from the beginning of the mesa strongly suggests that mesas are not amplification artefacts, but are rather true indications of the binding of FoxA1 to the chromatin, which is blocking the exonuclease from continuing to digest the DNA. The strand of the mesa is strongly correlated with the orientation of the motif: top-strand mesas have forward-oriented motifs, while bottom-strand mesas have reverse-complemented motifs. Figure 3C shows paired top- and bottom-strand mesas, with paired motifs in a palindromic orientation, overlapping by 3 bp. Additional examples of mesas on both strands are shown in Additional file 8: Figure S7. This pattern is relatively common, suggesting that there is a structural explanation for this observation, and indicating the presence of two FoxA1 proteins occupying the locus, one protecting each strand. Interestingly, a recent computational analysis of the ENCODE DNase I hypersensitivity-sequencing (DHS-seq) data predicts that the protein FoxA1 can bind forkhead motif-dimers as a homodimer [13]. They identify hundreds of forkhead-motif dimers in open regions of LNCaP cells. Using an *in silico* interaction prediction based on the crystal structure of the DNA-binding domain of forkhead proteins [14], they show that the binding of two forkhead proteins on a motif-dimer is structurally possible, a hypothesis supported by our experimental approach.

## Conclusions

We provide a protocol for ChIP-exo based on the commonly used Illumina sequencing platform. As ChIP-seq provided a substantial improvement over ChIP-chip in the accuracy of peak calling and ability to distinguish nearby binding sites [3,15], our data strongly suggest that ChIP-exo outperforms ChIP-seq in the ability to discriminate nearby peaks and small peaks. In addition, it can reveal insights into the patterns of transcription factor binding to the DNA, including the prediction of transcription factor dimer binding. We also show for the first time that ChIP-exo is feasible in primary tissue such as mouse liver. We believe that the ChIP-exo technology can help characterise the architecture of the *cis*-regulatory elements, particularly with regards to highlighting the cooperativity between transcription factors.

### Data availability

All data are deposited in ArrayExpress with accession number E-MTAB-1827. Figure 3A includes mouse FoxA1 ChIP-seq data deposited under the ArrayExpress accession numbers E-MTAB-223 [8] and E-MTAB-1414 [16].

## Materials and methods

### Biological material

MCF7, MDA-MB231, MDA-MB453, LNCaP and ZR75-1 human cell lines were obtained from ATCC and grown in DMEM or RPMI (LNCaP and ZR75-1) supplemented with 10 % FBS. The liver material was isolated from three adult (4 months) C57/BL6 males obtained from Cancer Research UK Cambridge Institute. The investigation was approved by the ethics committee and followed the Cambridge Institute guidelines for the use of animals in experimental studies under home office license PPL80/2197.

### Antibodies

The antibodies used for the ChIP-exo were anti-FoxA1 (ab5089) from Abcam, anti-ER (sc-543) and anti-FOXA2 (sc-6554) from Santa Cruz Biotechnologies.

### Chromatin immunoprecipitation sequencing

The ChIPs were performed as described previously [17], using 10 ug of anti-ER antibody (Santa Cruz, ref. sc-543). The ChIP-seq and the input libraries were prepared using the TruSeq ChIP Sample Prep Kit (Illumina, ref. IP-202-1012).

### Chromatin immunoprecipitation-exonuclease on Illumina sequencing platform

The main differences between our protocol and the Pugh protocol [5] are the oligonucleotides sequences, different washing buffer, the use of magnetic beads and the PCR mix. For the *lambda* exonuclease digestion, we have tested the Pugh conditions (10 units for 30 min) and a higher concentration (50 units for 1 h) on an ER ChIP-exo conducted in MCF-7 cells (Additional file 9). We found no significant difference in peak width with increased exonuclease concentration.

The cross-linking, cell lysis and sonication are done as described previously [17]. Each ChIP is done using 10 ug of antibody and 50 uL of Protein A or G magnetic beads (Invitrogen, Dynabeads). After the overnight ChIP on rotator at 4°C, the supernatant is removed and the beads are washed six times in 1 mL of RIPA buffer (50 mM HEPES pH 7.6; 1 mM EDTA; 0.7% Na-Deoxycholate; 1% NP-40; 0.5 M LiCL) in a 2 mL microfuge tube, followed by two washes in 1 mL of Tris HCl pH 8. The beads then undergo five successive incubations in a 2 mL tube agitated at 900 rpm in a thermomixer as followed:

1) End polishing: 1 mM ATP, 100 uM dNTP, 15 U T4 DNA polymerase, 5 U Klenow DNA polymerase, 50 U T4 PolyNucleotide Kinase, in 100 uL 1× NEBuffer 2 (50 mM NaCl, 10 mM Tris–HCl, 10 mM MgCl2, 1 mM DTT, pH 7.9) at 30°C for 30 min.

2) Ligation of the P7 exo-adapter: 1 mM ATP, 150 pmol P7 exo-adapter, 2000 U T4 DNA ligase, in 100 uL 1× NEBuffer 2 at 25°C for 60 min.

3) Nick repair: 150 uM dNTP, 15 U phi29 DNA polymerase in 100 uL 1× phi29 reaction buffer (50 mM Tris–HCl pH 7.5, 10 mM MgCl2, 10 mM (NH4)2SO4, 1 mM DTT, pH 7.5) at 30°C for 20 min.

4) *Lambda* exonuclease digestion: 10 U *Lambda* exonuclease in 100 uL 1× NEB *Lambda* exonuclease buffer (67 mM Glycine-KOH, 2.5 mM MgCl2, 50 μg/mL BSA, pH 9.4) at 37°C for 30 min.

5) RecJf exonuclease digestion: 30 U RecJf exonuclease in 100 uL NEBuffer 2 at 37°C for 30 min.

The beads are washed two times in 1 mL RIPA buffer and two times in 1 mL Tris HCl pH 8 after every incubation. All the incubations (1 to 5) are done so that the maximum concentration of DTT is 1 mM to avoid the elution of the ChIP material.

(6) Elution and reverse cross-linking: the beads are incubated with 100 ug of Proteinase K in 200 uL of elution buffer (50 mM Tris HCl pH 8; 10 mM EDTA; 1% SDS) overnight at 65°C. The 200 uL of supernatant is transferred to a new tube and diluted in 200 uL TE (10 mM Tris, 1 mM EDTA, pH 7.4). The DNA is purified using phenol-chloroform-isoamyl alcohol extraction followed by ethanol precipitation. The resulting DNA pellet is dissolved in 20 uL water. The DNA can be stored at this step at −20°C.

(7) P7 primer extension: the 20 uL of DNA is denaturated 5 min at 95°C, then mixed with 5 pmol of the P7 primer and incubated in 50 uL 1× NEB Phi29 reaction buffer for 5 min at 65°C and 2 min at 30°C in a thermocycler. After the addition of 10 U Phi29 DNA polymerase and 200 uM dNTP, the mix is incubated 20 min at 30°C and then 10 min at 65°C. The DNA is purified using AMPure beads (1.8 volume) and eluted in 20 uL of resuspension buffer (Tris-Acetate 10 mM pH 8).

(8) Ligation of the P5 exo-adapter: the 20 uL of DNA is mixed with 15 pmol of the P5 exo-adapter, 2,000 U T4 DNA ligase and incubated in 50 uL 1× NEB T4 DNA ligase buffer for 60 min at 25°C and then 10 min at 65°C. The DNA is purified using AMPure beads (1.8 volume) and eluted in 20 uL of resuspension buffer (Tris-Acetate 10 mM pH 8).

(9) PCR amplification: the DNA sample is amplified using 0.5 uM of the universal reverse PCR primer and the forward PCR primer containing the index sequence of choice in 50 uL 1× NEBNext High-Fidelity PCR Master Mix (New England Biolabs, M0541). The number of PCR cycles is 13 to 18, depending on the ChIP efficiency. The PCR product is purified using AMPure beads (1.8 volume) and eluted in 20 uL of resuspension buffer (Tris-Acetate 10 mM pH 8).

(10) Gel-size selection: 200 to 300 bp PCR product is purified from a 2% agarose gel using MinElute Gel Extraction Kit (Qiagen) and eluted in 20 uL of elution buffer.

(11) Illumina sequencing: the library is quantified using the KAPA library quantification kit for Illumina sequencing platforms (KAPA Biosystems, KK4824) and sequenced on a MiSeq, GAII or HiSeq following the manufacturer’s protocol.

### Oligonucleotides

The oligonucleotides were synthesised by Sigma-Aldrich and purified by HPLC (sequences in Table 3). The P7 exo-adapter and the P5 exo-adapter were obtained in mixing the couple of complement oligonucleotides in an Annealing Buffer (10 mM Tris pH 8, 50 mM NaCl, 1 mM EDTA) and annealed by heating 5 min at 95°C then let cool slowly to room temperature. The oligonucleotides designed for ChIP-exo are adapted from the oligonucleotide sequences © 2007–2012 Illumina, Inc. All rights reserved. Derivative works created by Illumina customers are authorised for use with Illumina instruments and products only. All other uses are strictly prohibited.

### Computational analysis

After sequencing, reads were aligned to human genome version GRCh37 (hg19) using BWA version 0.7.5a, and BAM-formatted files were created using samtools version 0.1.18. Reads with mapping quality less than 5 were discarded; reads overlapping ENCODE’s ‘signal artefact’ regions were also discarded [1]. These regions show significant signal for all or most transcription factors and histone marks, across many cell lines, so are presumed to be artefactual.

#### ER and related factors

Example peaks in figures, showing the top- and bottom-strand reads in red and blue, respectively, were made by splitting the reads into top- and bottom-strand, then generating bedgraph files for both using custom software, converting them to bigwig using UCSC’s ‘bedGraphToBigWig’ software [18], making overlay track hubs for the two strands, and viewing them with the UCSC genome browser [19,20].

Triplicates of ER ChIP-exo and -seq were converted to consensus peaks by identifying locations in which all three replicates had summits within 100 bp, and choosing the strongest peak’s summit as the true summit. Overlaps between the consensus summit sets were calculated by considering peaks to overlap if their summits were within 100 bp, using the BEDTools package [21]. Read density around summits was calculated using custom software; plots were made using the ‘ggplot2’ R package [22,23].

Motif frequency (Additional file 6: Figure S5) was calculated by scanning for motifs within 100 bp of peak summits using FIMO [24], then counting the number of regions with at least one motif with *p*-value <0.0025. Motif strength was calculated the same way, except taking the strongest motif (lowest *p*-value) in each region as the defining one.

#### Motif density analysis

To calculate the densities of ER, FoxA1 and GATA3 motifs (Figure 2D and 2E), we analysed the genomic sequence 250 bp upstream and downstream of ER summits. Motif occurrences were found using FIMO and TRANSFAC motifs at a *p*-value threshold of 10^-3^[24]. The number of motif occurrences at each position relative to the ER summit was summed and normalised by the total number of motif occurrences. Motif density profiles were smoothed using a weighted moving average in 20 bp windows where weights are shaped as an isosceles triangle and the central point is given the maximum weight. The characteristic width of the ER motif density was computed by finding the width of the region where the density is greater than 1 in 500 base pairs. Matrices used: ER: M01801, FoxA1: M00724, GATA3: M00351.

#### FoxA1 mesas and related analyses

Figure 3B shows the occurrence of forkhead motifs around positions where the read depth on one strand increases by 100 reads between one nucleotide and the next. Some such positions lack motifs; conversely some positions with smaller increase in read depth have correctly positioned motifs. Three parameters may be varied: increase in read depth, presence of motif with some *p-*value, and the stringency of the positioning (9 bp is most common, but 8 bp and 10 bp also occur with some frequency). A range of parameter combinations was tried (data not shown); in the end, a true mesa is defined as one with a read depth increase of at least 30 bp and a forkhead motif with the correct orientation relative to the mesa, a *p-*value < = 0.0025, and a motif position of 8 to 10 bp from the read depth increase. These values were chosen because they reflected reasonably clear inflection points in the plots of mesa occurrences as parameters changed. Peaks were classified as paired mesas if they had mesas on the top and bottom strands, with paired motifs in palindromic orientation and their trailing ends within 5 bp of each other.

## Abbreviations

ChIP: Chromatin immunoPrecipitation; ER: Estrogen receptor-*alpha*; FoxA1: Hepatocyte nuclear factor 3-*alpha*; FoxA2: Hepatocyte nuclear factor 3-*beta*; GWAS: Genome-wide association study; MACS: Model-based analysis of ChIP-Seq data; SNP: Single nucleotide polymorphism.

## Competing interests

The authors declare that they have no competing interests.

## Authors’ contributions

AAS, GDB and JSC conceived all experiments. All experimental work was conducted by AAS. All bioinformatics work was conducted by GDB, with help from JDC. The manuscript was written by AAS, GDB and JSC and was read and edited by JDC. All authors read and approved the final manuscript.

## Supplementary Material

Additional file 1: Table S1 Oligonucleotide sequences. **Table S2.** Multiplexed samples. **Table S3.** Samples used to assess peak accuracy. **Table S4.** ChIP efficiency (reads in peaks). **Table S5.** Samples used in mesa analysis. Click here for file

Additional file 2: Figure S1 Example of the *TFF1*/*TMPRSS3* locus showing the 12 ER ChIP-exo libraries performed in MCF-7 cells and efficiently demultiplexed after sequencing in one lane of HiSeq. The ChIP-exo signal is roughly the same between libraries. This indicates that the signal is not biased by the index number. Click here for file

Additional file 3: Figure S2 Illustration of the ChIP-seq and ChIP-exo Illumina libraries. **(A)** After the ChIP-exo library preparation, each ChIPed DNA fragment results in two library fragments: one with the P5 adapter ligated downstream of the exonuclease digestion-protected DNA and the other with the P5 adapter ligated upstream of it. In each case, the P7 adapter is ligated to the other extremity. The 36 bp single-end sequencing of the ChIP-exo library results in two overlapping populations of reads, one mapped on the top strand and the other mapped on the bottom strand. **(B)** After the ChIP-seq library preparation, each ChIPed DNA fragment is ligated to the P7 and P5 adapters on both sides. The 36 bp single-end sequencing of the ChIP-seq library results in two shifted populations of reads, one mapped on the top strand and the other mapped on the bottom strand. Click here for file

Additional file 4: Figure S3 Examples of two ER binding sites identified by triplicate ChIP-seq and ChIP-exo libraries. **(A)** ER peak located upstream of the *GREB1* gene. **(B)** ER peak located in the gene body of the *TMPRSS3* gene. Click here for file

Additional file 5: Figure S4 Examples of four ER binding sites called by MACS via ChIP-seq or ChIP-exo. Click here for file

Additional file 6: Figure S5 Motifs: ChIP-exo *versus* ChIP-Seq. **(A)** ER motif frequency in different types of peaks. **(B)** Motif *p*-value in different types of peaks. Click here for file

Additional file 7: Figure S6 Venn diagrams showing the reproducibility of peaks called in three replicates of ER ChIP-seq and ChIP-exo performed in MCF-7 cells. Click here for file

Additional file 8: Figure S7 Conservation of mesas across cell lines. This figure shows three FoxA1 paired mesas identified in MCF-7 (ER + breast cancer cells), LNCaP (AR + prostate cancer cells), MDA-MB-453 (ER- AR + breast cancer cells) and ZR75-1 (ER + breast cancer) cell lines. The third mesa is missing in LNCaP cells. Click here for file

Additional file 9: Figure S8 Peak width under different ChIP-exo digestion conditions, compared with two replicates of ChIP-seq. The *lambda* exonuclease digestion was tested using the Pugh’s condition (10 units for 30 min) or using a greater concentration (50 units for 1 h) on an ER ChIP-exo conducted in MCF-7 cells. Click here for file
