# Supplementary figures and images for: Development of an Illumina-based ChIP-exonuclease method provides insight into FoxA1-DNA binding properties
Source: Genome Biol. 2013 Dec 27;14(12):R147. doi: 10.1186/gb-2013-14-12-r147 (PMC4053927; doi:10.1186/gb-2013-14-12-r147)

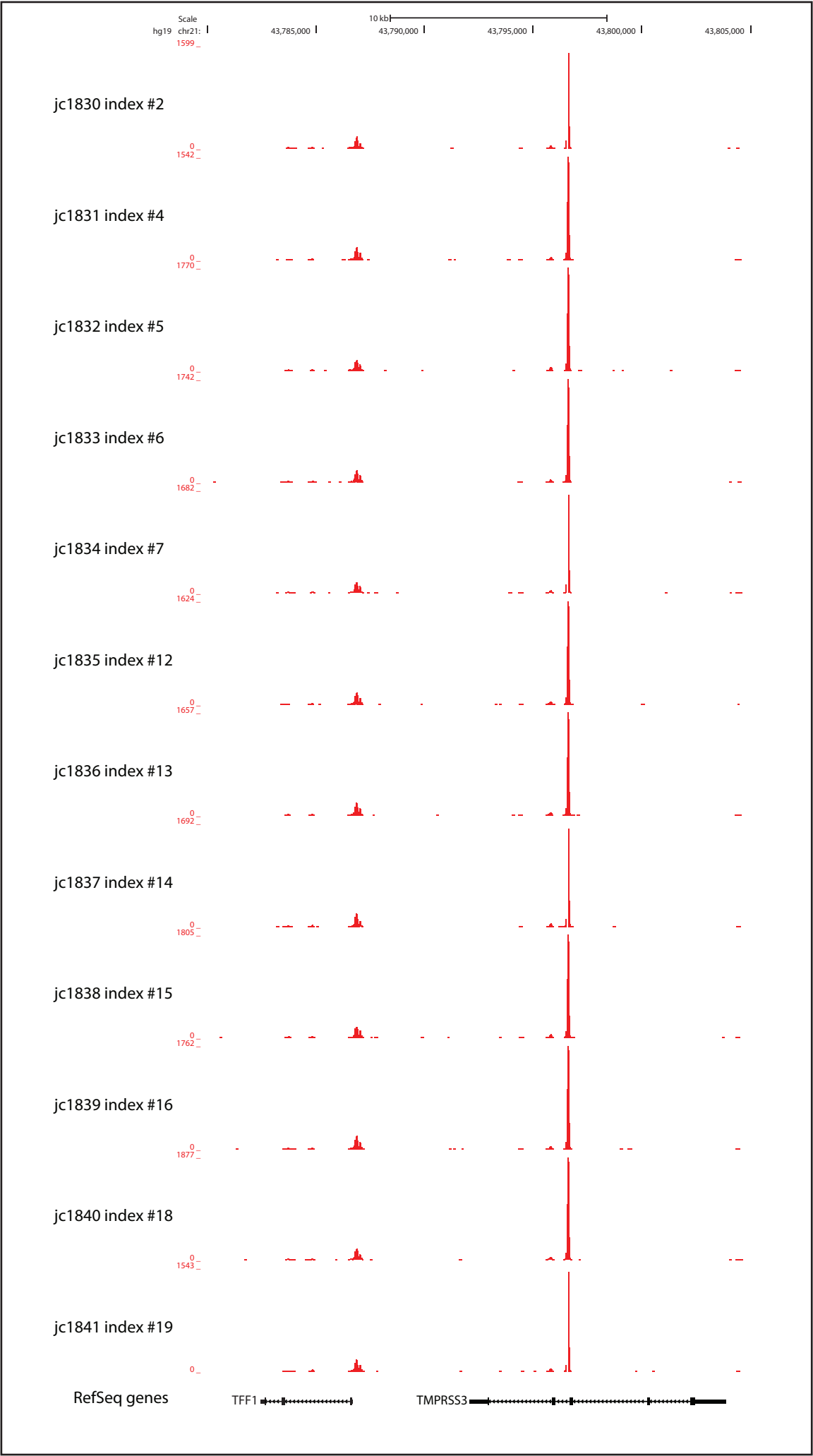

Supplement: Additional file 2: Figure S1 — Example of the TFF1/TMPRSS3 locus showing the 12 ER ChIP-exo libraries performed in MCF-7 cells and efficiently demultiplexed after sequencing in one lane of HiSeq. The ChIP-exo signal is roughly the same between libraries. This indicates that the signal is not biased by the index number. [file gb-2013-14-12-r147-S2.pdf]

A)

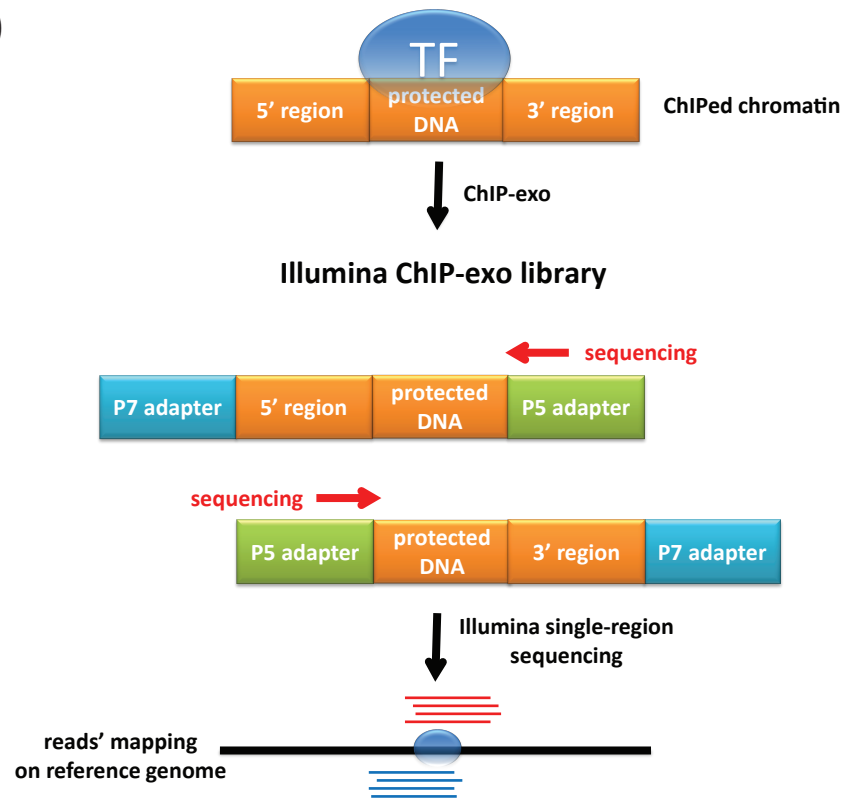

B)

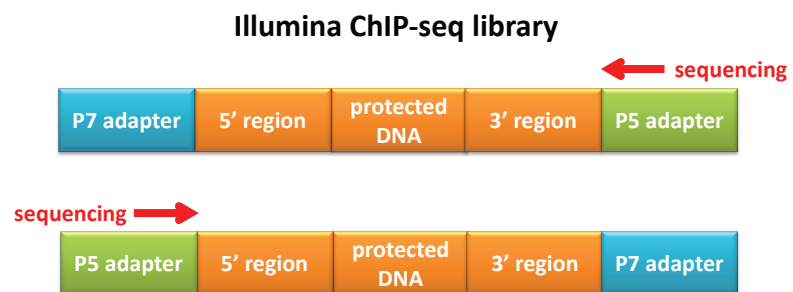

Supplement: Additional file 3: Figure S2 — Illustration of the ChIP-seq and ChIP-exo Illumina libraries. (A) After the ChIP-exo library preparation, each ChIPed DNA fragment results in two library fragments: one with the P5 adapter ligated downstream of the exonuclease digestion-protected DNA and the other with the P5 adapter ligated upstream of it. In each case, the P7 adapter is ligated to the other extremity. The 36 bp single-end sequencing of the ChIP-exo library results in two overlapping populations of reads, one mapped on the top strand and the other mapped on the bottom strand. (B) After the ChIP-seq library preparation, each ChIPed DNA fragment is ligated to the P7 and P5 adapters on both sides. The 36 bp single-end sequencing of the ChIP-seq library results in two shifted populations of reads, one mapped on the top strand and the other mapped on the bottom strand. [file gb-2013-14-12-r147-S3.pdf]

## A) ER binding site in *GREB1* enhancer

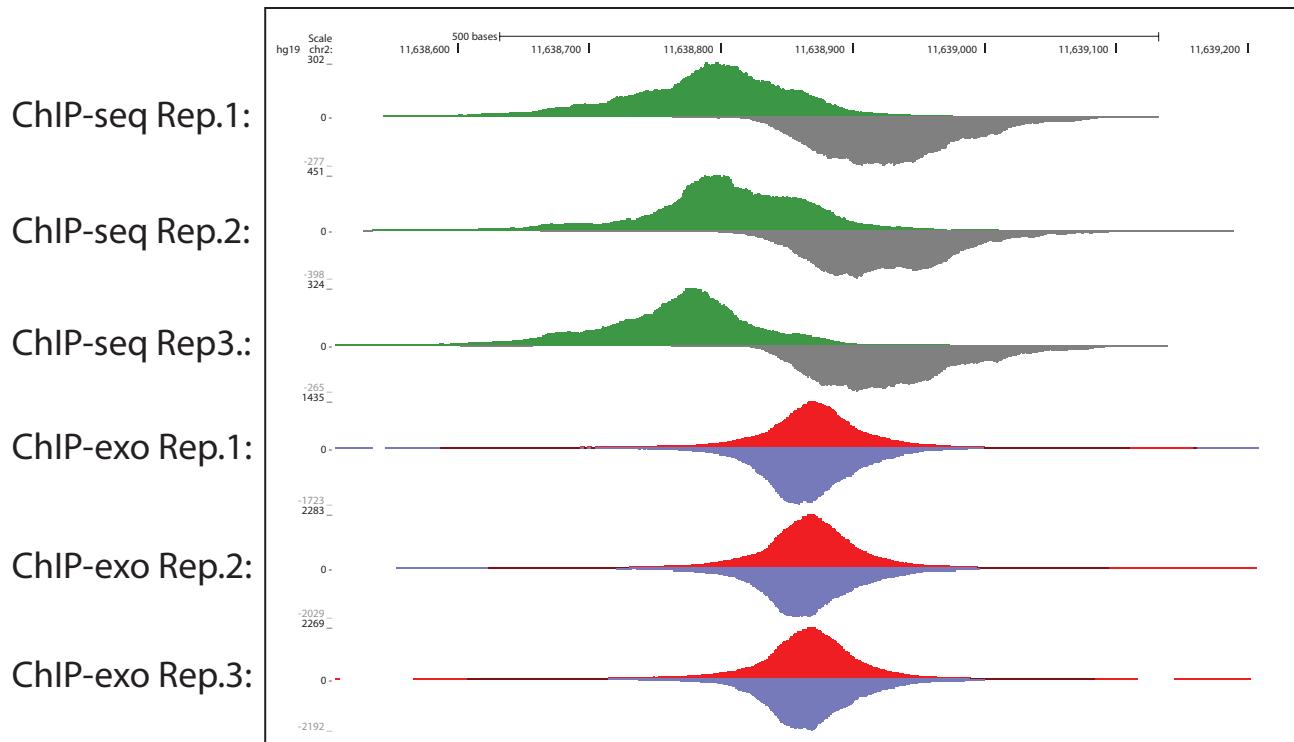

## B) ER binding-site in *TMPRSS3* gene body

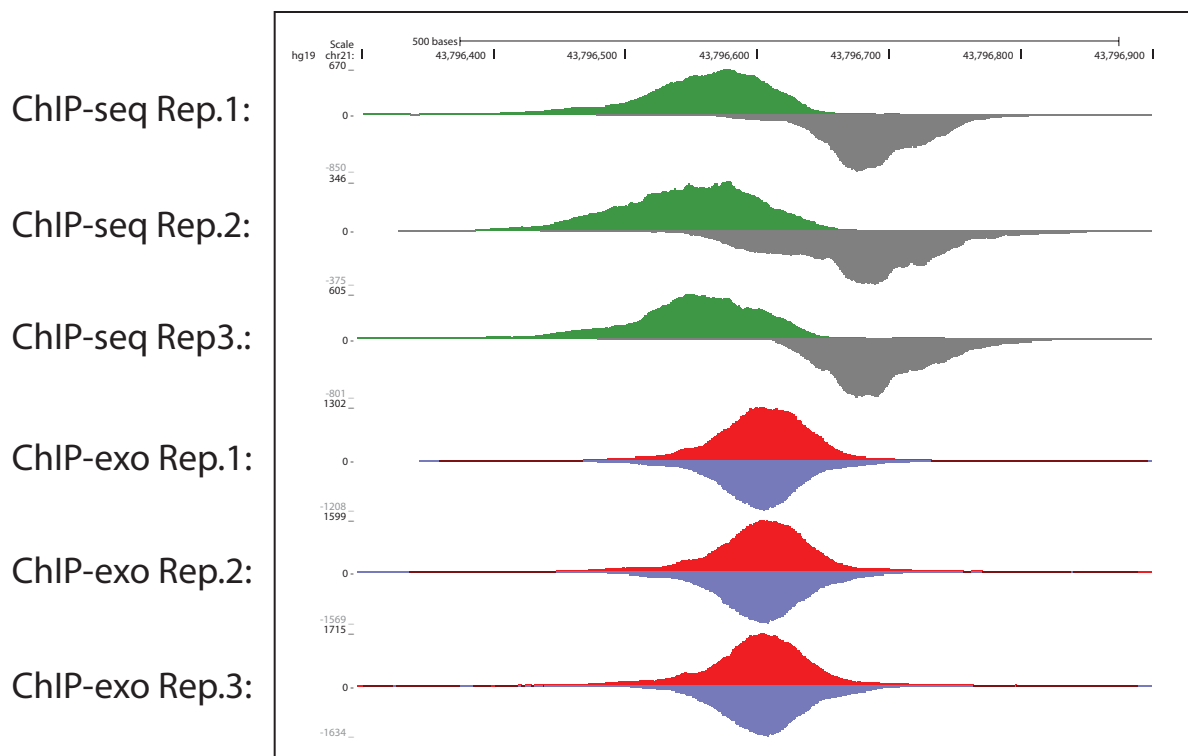

Supplement: Additional file 4: Figure S3 — Examples of two ER binding sites identified by triplicate ChIP-seq and ChIP-exo libraries. (A) ER peak located upstream of the GREB1 gene. (B) ER peak located in the gene body of the TMPRSS3 gene. [file gb-2013-14-12-r147-S4.pdf]

A)

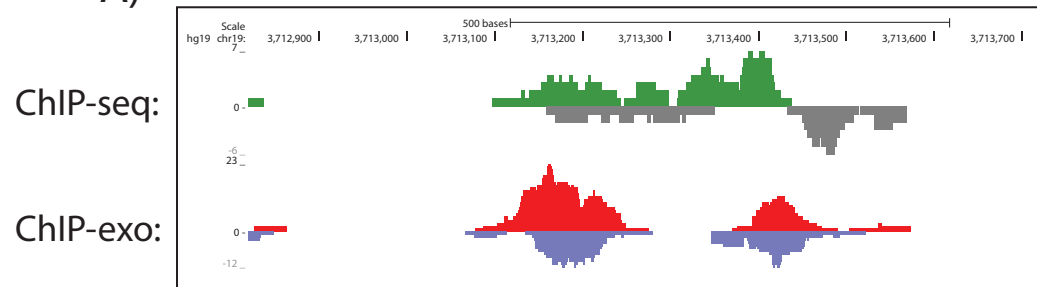

B)

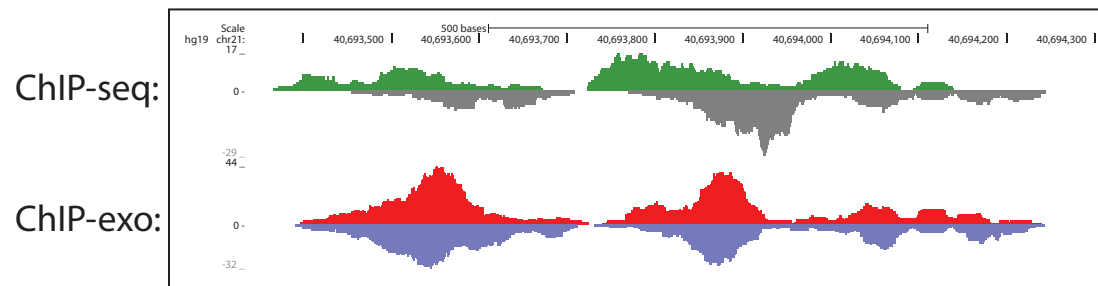

C)

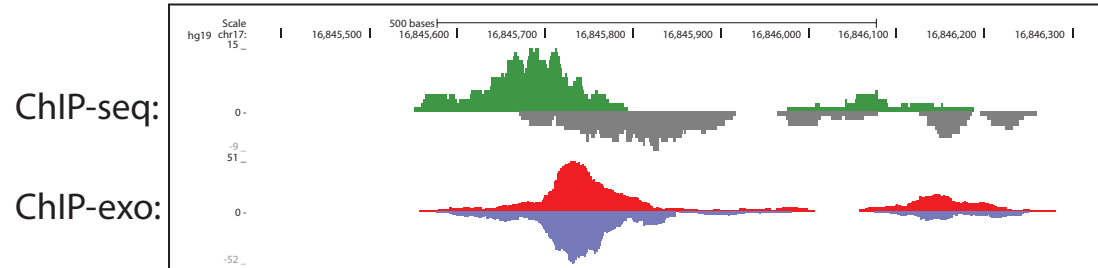

D)

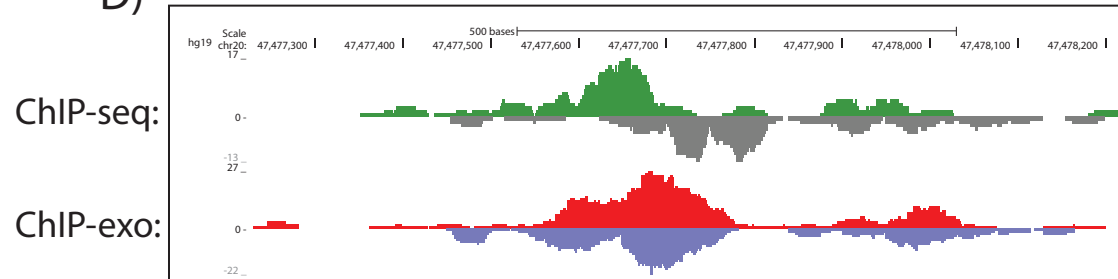

Supplement: Additional file 5: Figure S4 — Examples of four ER binding sites called by MACS via ChIP-seq or ChIP-exo. [file gb-2013-14-12-r147-S5.pdf]

A) Motif frequency by peak type

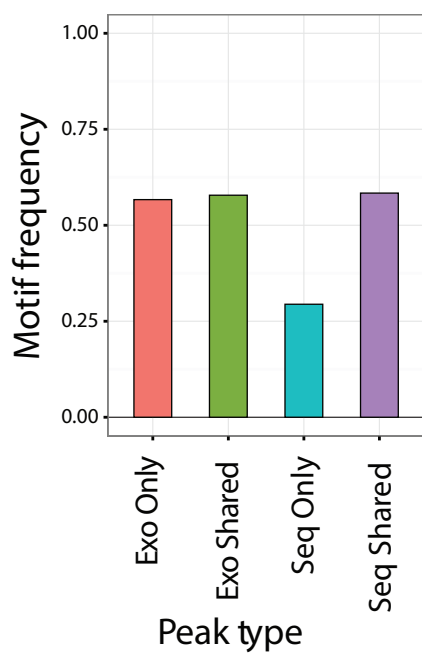

B) Motif strength ( $p$ -value)

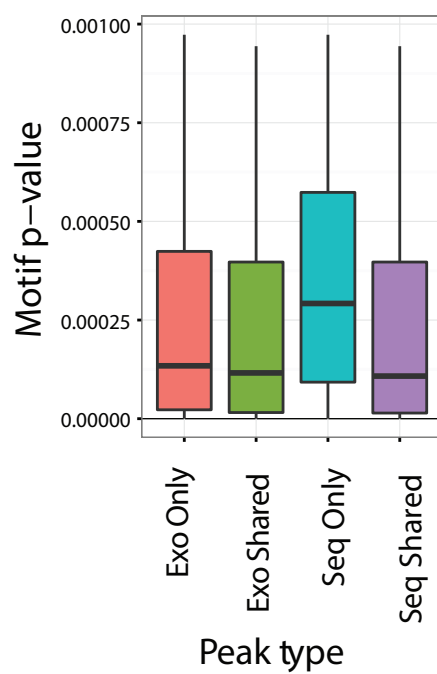

Supplement: Additional file 6: Figure S5 — Motifs: ChIP-exo versus ChIP-Seq. (A) ER motif frequency in different types of peaks. (B) Motif p-value in different types of peaks. [file gb-2013-14-12-r147-S6.pdf]

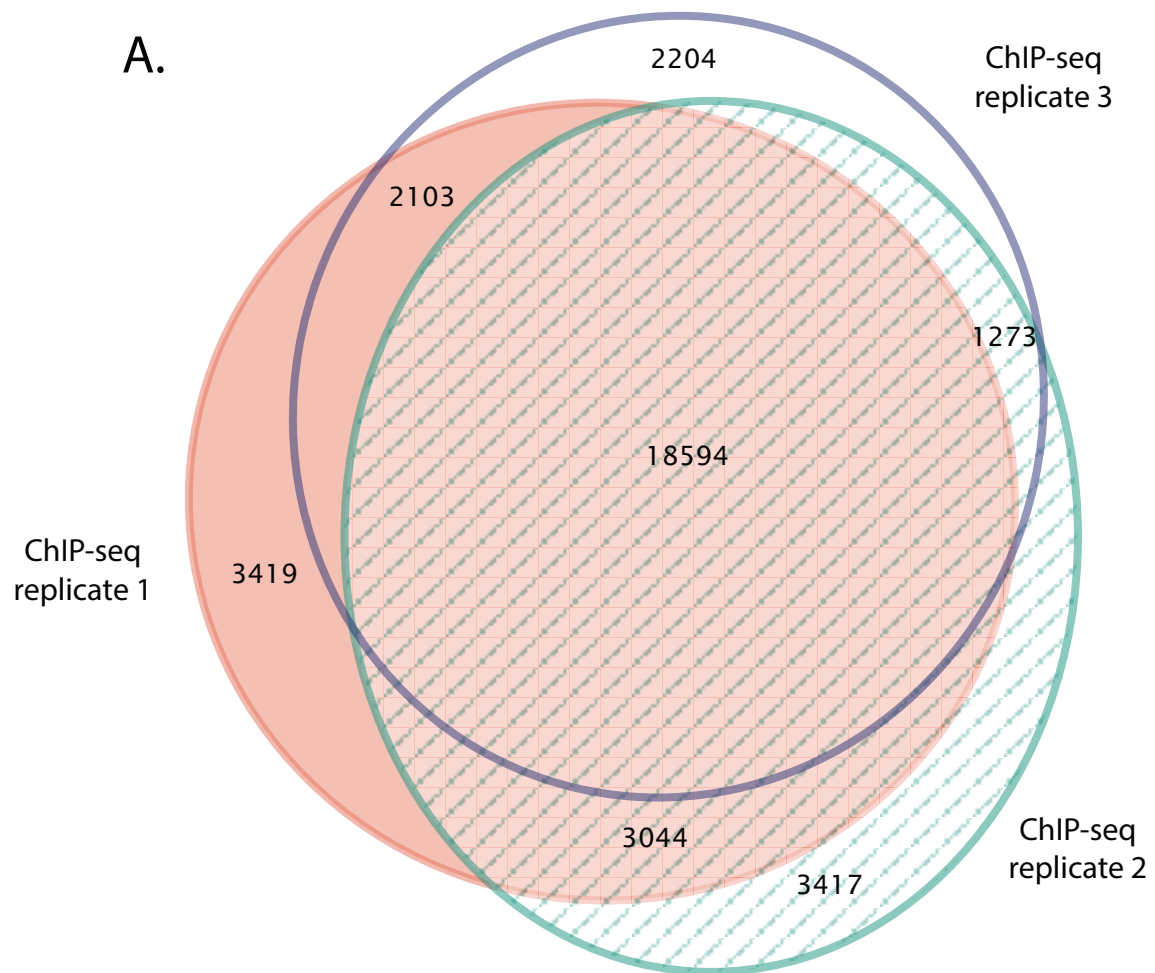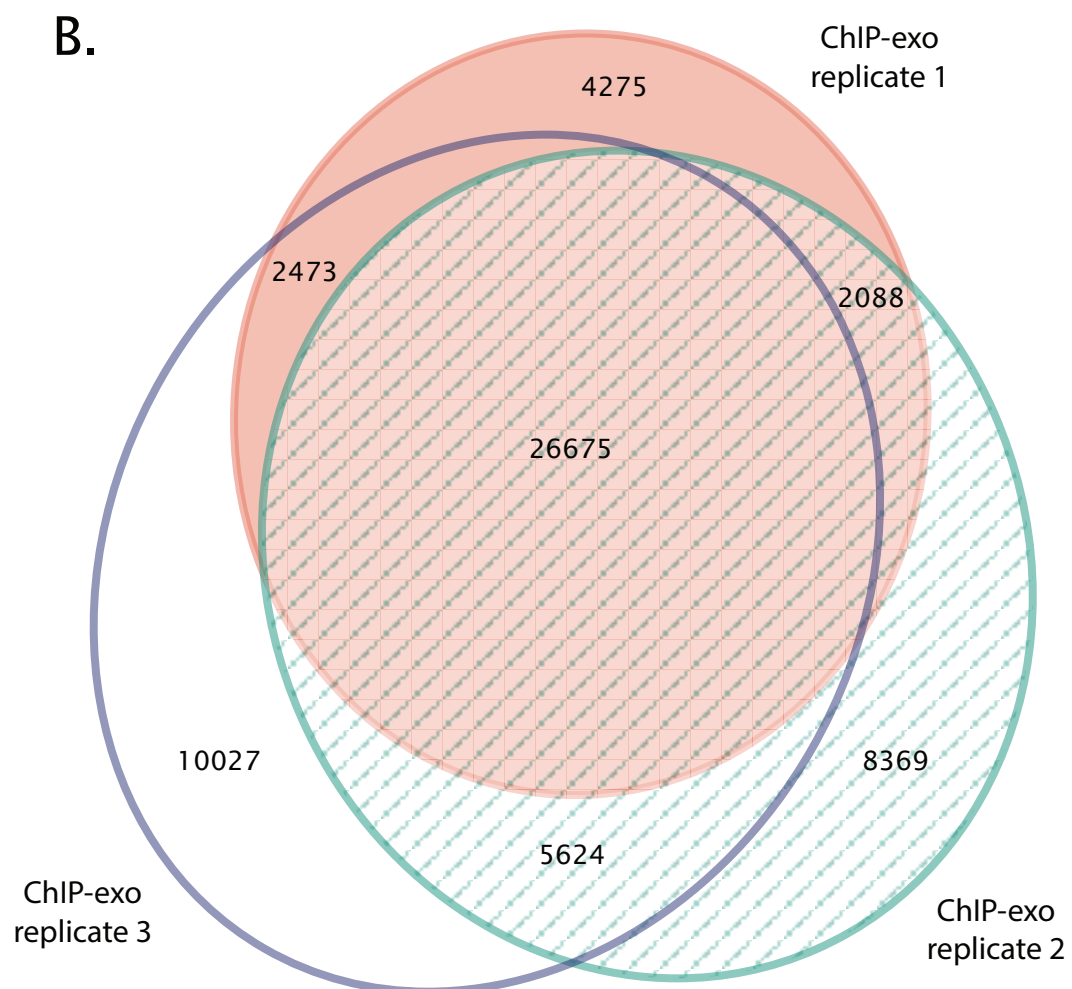

Supplement: Additional file 7: Figure S6 — Venn diagrams showing the reproducibility of peaks called in three replicates of ER ChIP-seq and ChIP-exo performed in MCF-7 cells. [file gb-2013-14-12-r147-S7.pdf]

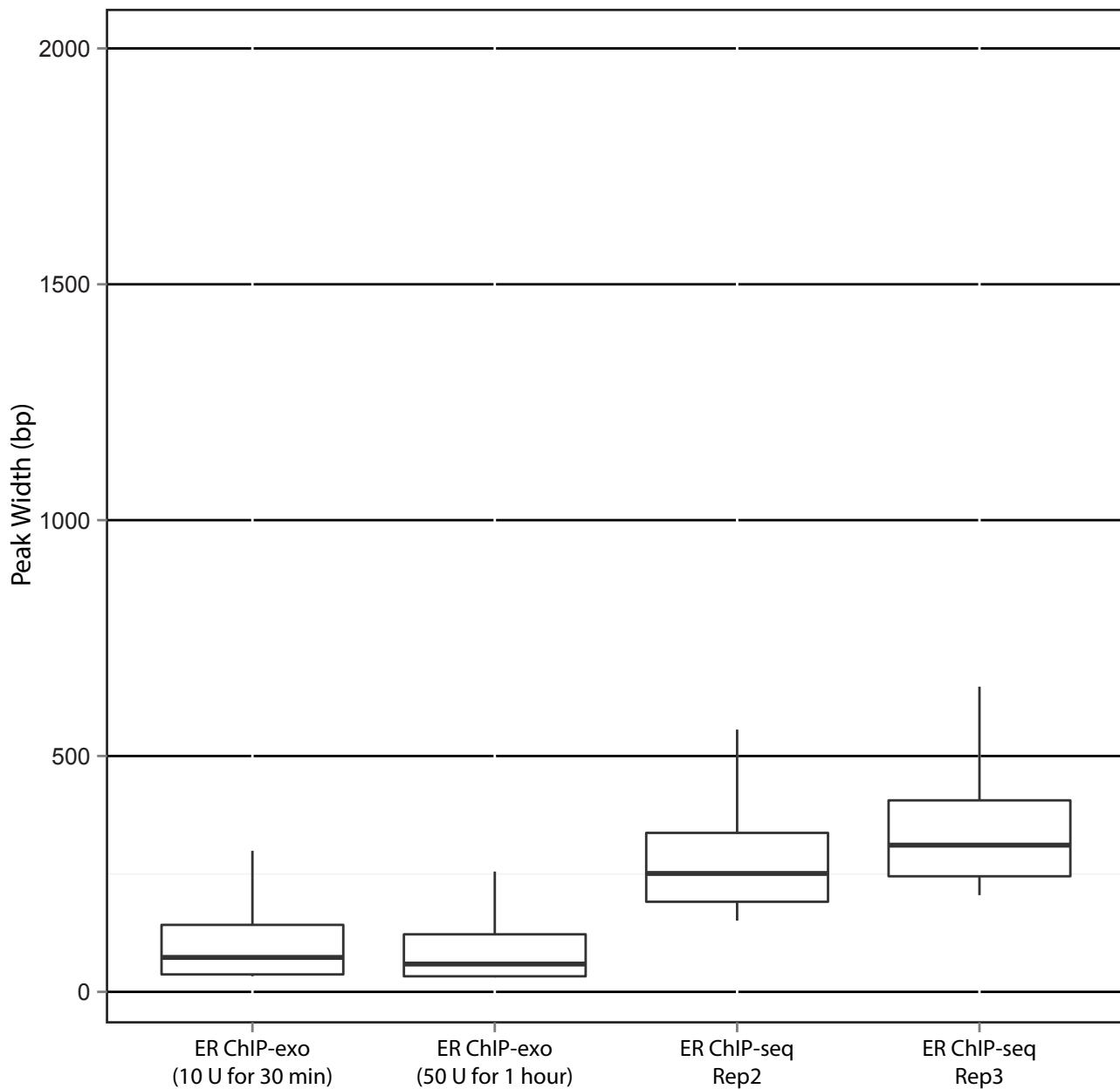

Supplement: Additional file 9: Figure S8 — Peak width under different ChIP-exo digestion conditions, compared with two replicates of ChIP-seq. The lambda exonuclease digestion was tested using the Pugh’s condition (10 units for 30 min) or using a greater concentration (50 units for 1 h) on an ER ChIP-exo conducted in MCF-7 cells. [file gb-2013-14-12-r147-S9.pdf]
