# Supplementary material for: Development of an Illumina-based ChIP-exonuclease method provides insight into FoxA1-DNA binding properties
Source: Genome Biol. 2013 Dec 27;14(12):R147. doi: 10.1186/gb-2013-14-12-r147 (PMC4053927; doi:10.1186/gb-2013-14-12-r147)
Supplement: Additional file 8: Figure S7 — Conservation of mesas across cell lines. This figure shows three FoxA1 paired mesas identified in MCF-7 (ER + breast cancer cells), LNCaP (AR + prostate cancer cells), MDA-MB-453 (ER- AR + breast cancer cells) and ZR75-1 (ER + breast cancer) cell lines. The third mesa is missing in LNCaP cells. [file gb-2013-14-12-r147-S8.pdf]

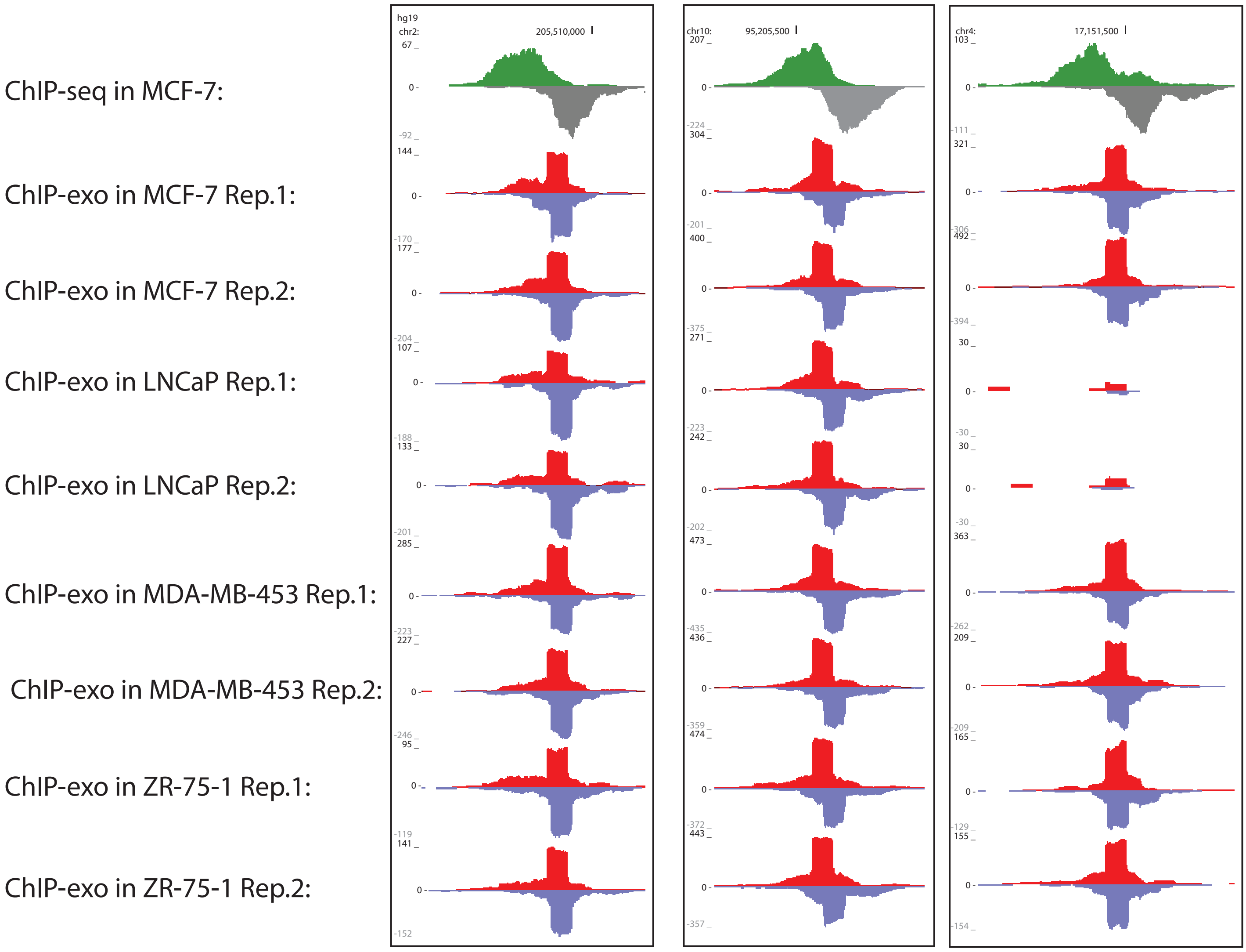

ChIP-seq in MCF-7:
ChIP-exo in MCF-7 Rep.1:
ChIP-exo in MCF-7 Rep.2:
ChIP-exo in LNCaP Rep.1:
ChIP-exo in LNCaP Rep.2:
ChIP-exo in MDA-MB-453 Rep.1:
ChIP-exo in MDA-MB-453 Rep.2:
ChIP-exo in ZR-75-1 Rep.1:
ChIP-exo in ZR-75-1 Rep.2:
hg19
chr2:
205,510,000
chr10:
95,205,500
chr4:
17,151,500
